# Inhibitory effect of reduced graphene oxide-silver nanocomposite on progression of artificial enamel caries

**DOI:** 10.1590/1678-7757-2018-0042

**Published:** 2018-12-10

**Authors:** Ruixue Wu, Qi Zhao, Shushen Lu, Yuanxiang Fu, Dongsheng Yu, Wei Zhao

**Affiliations:** 1 Sun Yat-sen University, Guangdong Provincial Key Laboratory of Stomatology, Hospital of Stomatology, Guanghua School of Stomatology, Guangzhou, China.; 2 The First Affiliated Hospital Of Hubei University Of Science And Technology, Xianning Central Hospital, Xianning, China.; 3 School of Chemistry and Chemical Engineering, Sun Yat-sen University, Guangzhou, China.

**Keywords:** Graphene, Silver, Nanoparticles, Antibacterial agents, Dental caries

## Abstract

The use of antimicrobial agents is an efficient method to prevent dental caries. Also, nanometric antibacterial agents with wide antibacterial spectrum and strong antibacterial effects can be applied for prevention of dental caries.

**Objectives:**

The aim of this study was to evaluate the inhibitory effect of reduced graphene oxide-silver nanoparticles (rGO/Ag) composite on the progression of artificial enamel caries in a *Streptococcus mutans* biofilm model.

**Material and Methods:**

Enamel specimens from bovine incisors were divided into eight treatment groups (n = 13), as follows: group 1 was inoculated with *S. mutans* grown in Brain Heart Infusion containing 1% sucrose (1% BHIS), as negative control; groups 2-4 were inoculated with *S. mutans* grown in the presence of different rGO/Ag concentrations (0.08, 0.12, 0.16 mg/mL) + 1% BHIS; group 5-7 were inoculated with *S. mutans* grown in the presence of different agents (0.16 mg/mL reduced graphene oxide, 0.16 mg/mL silver nanoparticles, 10 ppm NaF) + 1% BHIS; group 8 was mixed with 1% BHIS, without inoculation. Artificial enamel carious lesions were produced by *S. mutans* biofilm model for 7 days. Confocal laser scanning microscopy and atomic force microscopy were used to analyze roughness and morphology of the enamel surface. Polarized light microscopy and confocal laser scanning microscopy were employed to measure the lesion depth and the relative optical density (ROD) of the demineralized layer.

**Results:**

Compared with the control groups, the rGO/Ag groups showed: (a) reduced enamel surface roughness; (b) much smoother and less eroded surfaces; (c) shallower lesion depth and less mineral loss.

**Conclusion:**

As a novel composite material, rGO/Ag can be a promising antibacterial agent for caries prevention.

## Introduction

Dental caries is a common chronic i nfectious oral disease affecting both adults and children. Extensive efforts in preventing caries through reduction of sucrose intake, enhancement of public awareness, and administration of fluoride have led to a decline in caries prevalence, ^1^ but it is still a major public health problem worldwide. Cariogenic bacteria are the known risk factor for dental caries. Consequently, application of antibacterial agents to inhibit biofilm accumulation over tooth surface is an innovative method for dental caries prevention. ^2^ However, the problem of antibiotic resistance hinders the application of such drugs. In recent years, metallic nanomaterials with wide antibacterial spectrum and strong antibacterial effects have been drawing great attention and have gradually been applied in the field of dentistry. ^3^

Graphene, as one atom-thick sheet of sp ^2^ -bonded carbon atoms packed in a dense two-dimensional honeycomb crystal, ^4^ is of the utmost interest due to its unique structure and valuable properties. Rich in oxygen-containing functional groups, graphene oxide nanosheets have good water dispersion and high cell compatibility. ^5^ Due to their advantageous properties, graphene oxide sheets have already been developed as substrates to carry inorganic antibacterial nanoparticles, such as silver (Ag), gold (Au), and titanium oxide (TiO_2_). ^6^ ^,^ ^7^ Silver nanoparticles (AgNPs), as broad-spectrum antimicrobial agents, have been used in many fields, such as infection treatment and production of dental materials. ^8^ However, the antibacterial properties of AgNPs could be diminished or even totally lost due to their strong tendency to self-aggregation. ^9^ Graphene oxide can effectively disperse AgNPs in water by acting as a substrate to anchor nanoparticles, thus improving the antibacterial property of AgNPs. As novel antibacterial systems, AgNPs assembled on graphene oxide nanosheets are increasingly attracting researchers’ interests. ^10^ Cai, et al. ^11^ (2012) reported that reduced graphene oxide-silver (rGO/Ag) composite have superior chemical stability and excellent antibacterial activity. Tang, et al. ^12^ (2013) demonstrated that silver nanoparticles assembled on graphene oxide sheets showed outstanding antibacterial effects against Gram-positive *Staphylococcus aureus* and Gram-negative *Escherichia coli.* Peng, et al. ^13^ (2017) compared the antibacterial activity of reduced graphene-silver nanoparticles (R-GNs/Ag), reduced graphene, and AgNPs; the results indicated that R-GNs/Ag composite exhibited the highest antibacterial action. Therefore, applying rGO/Ag as novel antibacterial agents in dental caries is promising, and the anticariogenic potential of this novel agent in combatting microbial biofilm-induced artificial caries remains as a matter to be investigated.

In this study, the *Streptococcus mutans* biofilm-induced caries model was used to evaluate the inhibitory effect of rGO/Ag composite on the progression of artificial enamel caries. The null hypothesis is that rGO/Ag composite has no effect on the progression of artificial enamel caries.

## Material and methods

### Preparation and characterization of rGO/Ag

GO powder was prepared using natural graphite flakes (325 mesh equals <44 micrometers, 99.8%) as raw material, according to a modified version of the Hummers method. ^14^ In brief, 150 mg of GO and 100 mg of polyvinylpyrrolidone (PVP) powder were dispersed in 100 mL of deionized water and homogenized by ultrasonication (Kunshan, Jiangsu, China, KQ-200, 100 W, 40 kHz) for 60 min. 150 mg of silver nitrate were added into the homogeneous solution and mixed for 30 min by magnetic stirring. 2 mL of hydrazine dilute solution was gradually added into the above solution, and the mixture was allowed to react for 120 min at 90°C. Following, the product was separated and washed by deionized water and ethanol for several times. Then the sample was freeze-dried for 10 hours and the rGO/Ag powder was collected.

Scanning electron microscopy (Quanta 400F-FEI, Eindhoven, Netherlands) and transmission electron microscopy (Tecnai G2 Spirit-FEI, Hillsboro, Oregon, USA) at an acceleration voltage of 120 kV were employed to characterize the morphology of the rGO/Ag composite. The silver content on the rGO/Ag composite was determined by thermogravimetric analyzer (TG209 F1-Netzsch, Free State of Bavaria, Germany). The analysis was carried out from room temperature to 800°C at a heating rate of 10°C/min under nitrogen flow rate of 20 mL/min. To study the silver ions (Ag^+^) released from rGO/Ag composite, the rGO/Ag composite was dispersed in deionized water under ultrasonic condition (Branson B5510E-DTH, Connecticut, USA, 100 W, 40 kHz) for 10 h. Then, the concentration of Ag^+^ was determined by inductively coupled plasma mass spectrometry (ICP-MS) (ICAP Qc-Thermo Fisher, MA, USA).

### Bacteria and culture conditions

*S. mutans* (UA159), which was grown in Brain Heart Infusion broth (BHI) at 37°C, under anaerobic conditions (80% N_2_; 10% H_2_; 10% CO_2_), was used in this study. To induce formation of *S. mutans* biofilm, 1% sucrose containing BHI (BHIS) was added.

### Antimicrobial effects of rGO/Ag on *S* . *mutans*

The minimum inhibitory concentration (MIC) and minimum biofilm inhibitory concentration (MBIC) of the rGO/Ag composite against the *S. mutans* were determined by the microdilution method. *S. mutans* at the concentration of 10 ^5^ -10 ^6^ CFU/mL was aliquoted in 96-well microtiter plates, and serially diluted with rGO/Ag (0.00125-0.64 mg/mL), and BHI/BHIS medium. After incubation under anaerobic conditions (80% N_2_; 10% H_2_; 10% CO_2_) at 37°C for 24 h, the MIC was determined as the lowest concentration of rGO/Ag that totally inhibited the bacterial growth, and the MBIC was determined as the lowest rGO/Ag concentration at which the formation of *S. mutans* biofilms was inhibited. ^15^ All the tests were performed in triplicate.

### Preparation of bovine enamel specimens

The study was conducted in full accordance with the Declaration of Helsinki of the World Medical Association and with local laws and regulations.

One hundred and four enamel specimens (5 mm × 4 mm × 3 mm) were prepared from freshly extracted sound incisor bovine teeth and stored in phosphate buffered saline at room temperature. Following teeth examination under a stereomicroscope (M205A, Leica, Germany), teeth with stains and erosions or microcracks on the enamel surfaces were excluded. Enamel surfaces were ground and polished sequentially using 500-grit, 800-grit, 1200-grit and 2000-grit silicon carbide sandpapers. Each specimen was sonicated in distilled water for 1 min to remove any residual abrasives, and an acid-resistant nail polish was applied on the surface of the specimen except for a 3 mm x 2 mm central region. Before the start of the experiment, the specimens were disinfected by being stored in a 75% alcohol solution for 12 hours and sterilized by ultraviolet light radiation for 2 hours.

### Formation of artificial caries

Enamel specimens were randomly assigned into eight groups (n=13), as follows: group 1 was inoculated with *S. mutans* grown in 1% BHIS, as negative control; groups 2-4 were inoculated with *S. mutans* grown in the presence of different concentrations of rGO/Ag (0.08, 0.12, 0.16 mg/mL) + BHIS; groups 5 and 6 were inoculated with *S. mutans* grown in the presence of different agents (0.16 mg/mL of reduced graphene oxide (rGO), 0.16 mg/mL of AgNPs) + BHIS; group 7 was inoculated with *S. mutans* grown in the presence of 10 ppm NaF + BHIS, as positive control; group 8 was mixed with BHIS without inoculation, as blank control.

Enamel specimens were used to assess the progression of caries through the *S. mutans* microbial biofilm-induced caries model. ^16^ For formation of salivary pellicles, all specimens were preserved in sterile artificial saliva (ChangFeng Technology, Dongguan, Guangzhou, China) at 37°C for 2 h. Immediately afterwards, specimens of groups 1–7 were carefully placed in a BHIS containing their respective agents and *S. mutans* (~10^6^ CFU/mL), and specimens of group 8 were kept in equal volumes of BHIS. All groups were incubated under anaerobic conditions at 37°C for 7 days, and the culture media were replaced daily with fresh BHIS containing the corresponding agents. After formation of enamel caries, the nail polish was carefully removed from each specimen using a surgical blade. All specimens were washed with deionized water to dislodge the attached biofilm and were stored in phosphate buffered saline at room temperature.

### Measurement of enamel surface roughness

The surface roughness of enamel was investigated by confocal laser scanning microscopy (LSM700-Carl Zeiss, Heidenheim, Germany) under 200x magnification to obtain three-dimensional topography images of the enamel surface. Areas of 300 μm × 300 μm squares on the enamel surface were analyzed to determine the average roughness (Ra) and root-mean-square roughness (Rq) for each specimen. Three measurements were randomly selected for each position of the enamel surface.

### Microstructural characterization of the enamel surface

An atomic force microscope (Dimension Fastscan Bio-Bruker, Karlsruhe, Germany) was operated in tapping mode using a nonconductive silicon nitride and with a scanning rate of 1 Hz. Three specimens were selected randomly from each group and kept in sterile artificial saliva at 37°C. Areas of 8 μm × 8 μm squares on the enamel rods were analyzed to map the enamel surface microstructure.

### Measurement of enamel lesion depth

Ten specimens were selected randomly from each group. They were longitudinally sectioned through the lesions with a diamond saw (Accotom-50, Struers, Copenhagen, Denmark) and polished sequentially using 500-grit, 800-grit, 1200-grit and 2000-grit silicon carbide sandpapers to obtain sections of approximately 100 μm in thickness (TegraMin-30, Struers, Copenhagen, Denmark). Polarized light microscopy (Axio Scope A1 pol-ZEISS, Heidenheim, Germany) was used to observe the morphology of enamel lesions and quantify the lesion depths.

### Assessment of the degree of demineralized layer

Following polarized light microscopy analysis, the same samples were immersed in a freshly prepared 0.1 mM rhodamine B (Guangfu Technology, Tianjin, China) solution for 1 hour, then rinsed 3 times with distilled water. Specimens were observed through confocal laser scanning microscopy (LSM780-Carl Zeiss, Heidenheim, Germany) at the excitation wavelength of 543 nm by a HeNe laser, and a 616 longpass filter at a 200x magnification. An image analysis system (Image Pro-Plus version 6.0) was used to measure the optical intensity and quantitatively analyze the digital images. The optical density (OD) is directly related to the porosity of the demineralized enamel. ^16^ ^,^ ^17^ The relative optical density (ROD) was calculated as OD_R_=OD_l_/OD_s_ × 100%, with OD_l_ being the OD of the enamel demineralized layer, and OD_s_ being the OD of the sound enamel tissue which was used as a blank control at the corresponding level (depth from the surface) of the specimen. OD_1_ and OD_s_ were measured at three different defined regions of the sample and the mean values were calculated.

### Statistical analysis

The statistical analysis was performed through the SPSS 20.0 software. Ra and ROD measurements were expressed as mean±standard deviation and analyzed by one-way analysis of variance (ANOVA) and *post-hoc* multiple comparison Student-Newman-Keuls (SNK) tests. The significance level was set at p<0.05.

## Results

### Characterization of rGO/Ag

**Figure 1:**
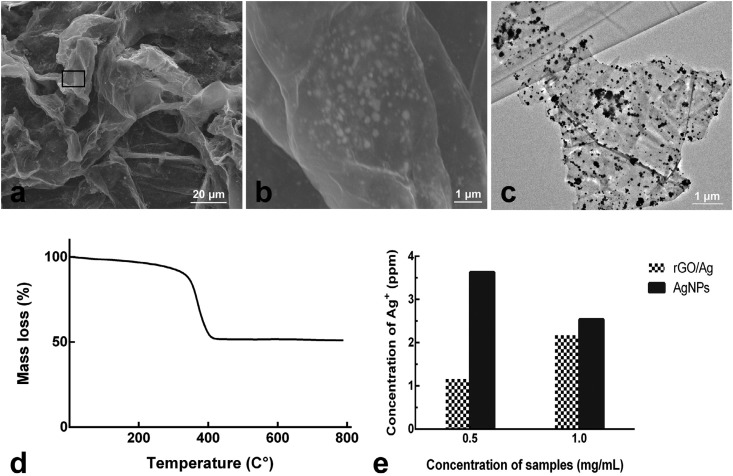
Characterization of the rGO/Ag composite. (a) scanning electron microscopy (SEM) image, (b) magnification of the area indicated by the black box in picture (a), (c) transmission electron microscopy (TEM) image, (d), thermogravimetric analysis (TGA) of rGO/Ag composite, (e) concentration of Ag+ released from the tested samples

Imaging analysis of the rGO/Ag composite by scanning electron microscope and transmission electron microscopy showed that rGO sheets effectively dispersed AgNPs by acting as substrates to anchor AgNPs nanoparticles, and that AgNPs attached to rGO sheets had no observed self-aggregation ( Figure 1 A-C).

The curve of the thermogravimetric analysis of the rGO/Ag composite is shown in Figure 1 D. The rGO/Ag composite exhibited mass loss at 327-400°C due to the decomposition of rGO; also, the total weight loss of rGO/Ag composite was about 49% at 800°C, indicating that the silver content on the rGO/Ag composite was 51%. According to the test result of Ag^+^ concentration ( Figure 1 E), rGO/Ag composite exhibited a lower concentration of Ag^+^ than that of AgNPs. It indicated that GO could efficiently control the release of Ag^+^, that and rGO/Ag composite displays a long term antibacterial effect.

### Antimicrobial activity of rGO/Ag on *S. mutans*

Values for MIC and MBIC of rGO/Ag against *S. mutans* UA159 were both estimated to be 0.16 mg/mL and 0.32 mg/mL. Therefore, to determine the effect of rGO/Ag on progression of artificial enamel caries, 0.08, 0.12, 0.16 mg/mL were chosen as test concentrations for the subsequent experiments.

### Enamel surface roughness

**Figure 2:**
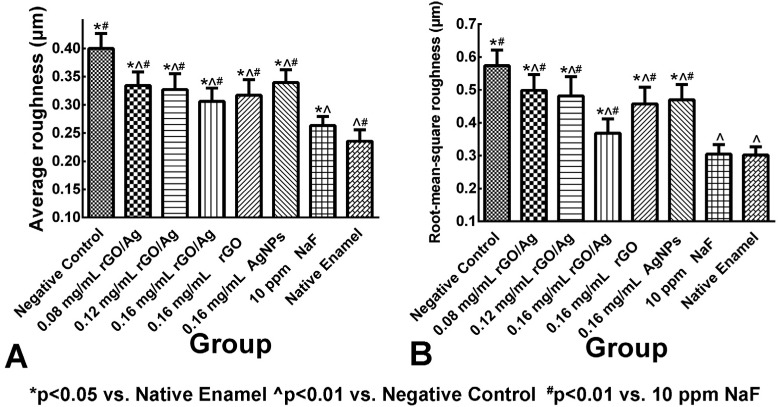
Graphical representation of the quantification results for enamel surface roughness of the teeth specimens in different treatment groups. (A) average roughness (Ra); (B) root-mean-square roughness (Rq). Ra and Rq values are expressed as mean±standard deviation (n=10)

Confocal laser scanning microscopy (CLSM) was used to obtain three-dimensional topography images. Apparent differences were observed on the surface roughness between the treatment and the control groups ( Figure 2 ). Based on data for the average surface roughness ( Figure 2 A), the Ra of native enamel and negative control groups were 0.24±0.02 μm and 0.40±0.03 μm, respectively, and this difference was statistically significant (p<0.05). The Ra of 0.08 mg/mL, 0.12 mg/mL and 0.16 mg/mL of groups treated with rGO/Ag was significantly reduced to 0.33±0.02 μm, 0.33±0.03 μm and 0.31±0.02 μm (p<0.05).

According to data on the root-mean-square roughness ( Figure 2 B), the Rq of the native enamel group was 0.30±0.03 μm and the negative control group was 0.57±0.05 μm. After treatment with rGO/Ag, the Rq of groups treated with 0.08 mg/mL and 0.12 mg/mL rGO/Ag was significantly reduced to 0.50±0.05 μm and 0.48±0.06 μm, respectively, and the one of the group treated with 0.16 mg/mL rGO/Ag (0.37±0.04 μm) showed the greatest reduction (p<0.05). Enamel specimens immersed in either 10 ppm NaF, or 0.16 mg/mL rGO or 0.16 mg/mL AgNPs solution, also had significantly reduced roughness, compared to the control group (p<0.01), with the respective Rq values being 0.30±0.03 μm, 0.46±0.05 μm, and 0.47±0.05 μm.

### Morphology of enamel surface

**Figure 3:**
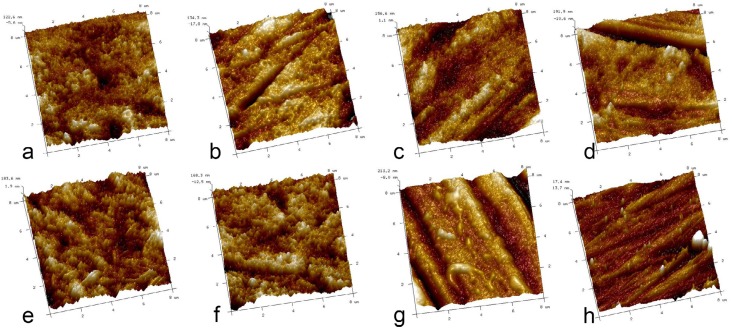
Atomic force micrographs of the enamel surface of specimens in different treatment groups. (a) negative control; (b) 0.08 mg/mL rGO/Ag; (c) 0.12 mg/mL rGO/Ag; (d) 0.16 mg/mL rGO/Ag; (e) 0.16 mg/mL rGO; (f) 0.16 mg/mL AgNPs; (g) 10 ppm NaF; (h) native enamel

The morphology of the enamel surface of samples from each treatment group is shown by atomic force microscopy (AFM) imaging in Figure 3 . The native enamel group displayed smooth and flat enamel surface with a few scratches and polishing debris. In the negative control group, surface defects such as rough areas, voids and cracks were visible. For the rGO/Ag groups, AFM images showed much smoother and less eroded surfaces, relative to the specimens in the other groups. In addition, this effect was dose-dependent, with the concentration of 0.16 mg/mL rGO/Ag having the greatest impact. In contrast, very rough surfaces, voids and cracks were still observed in the 0.16 mg/mL rGO and the 0.16 mg/mL AgNPs groups.

### Enamel lesion depth

**Figure 4:**
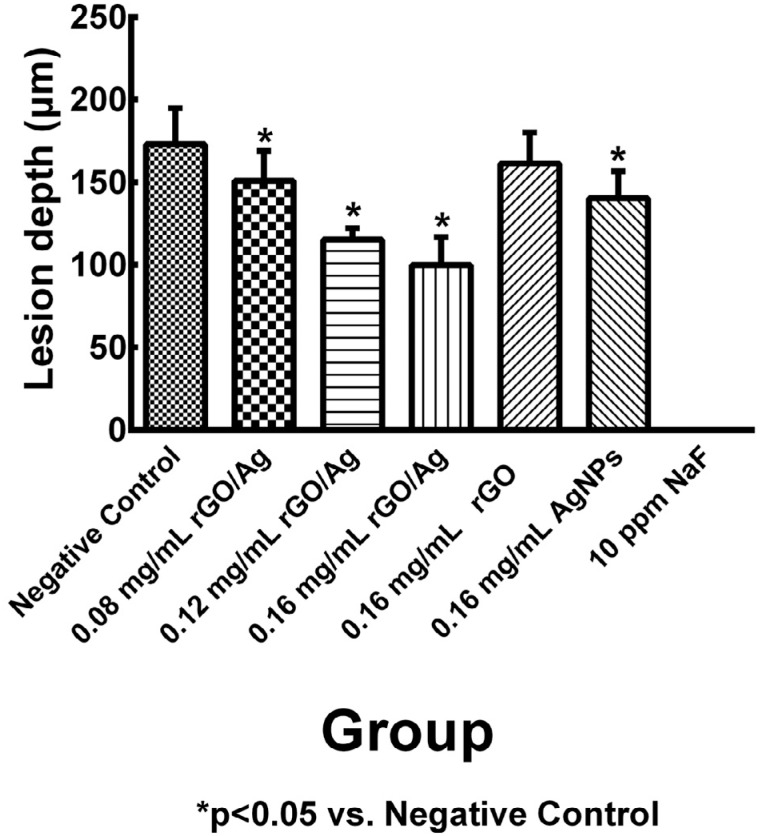
Graphical representation of the quantification results of the artificial enamel lesion depth of the teeth specimens in different treatment groups. (a) negative control; (b) 0.08 mg/mL rGO/Ag; (c) 0.12 mg/mL rGO/Ag; (d) 0.16 mg/mL rGO/Ag; (e) 0.16 mg/mL rGO; (f) 0.16 mg/mL AgNPs; (g) 10 ppm NaF; (h) native enamel. Lesion depth values are expressed as mean±standard deviation (n=10)

As shown in Figure 4 , it was apparent that the average lesion depths differed among the groups treated with rGO/Ag and negative control group. Base on data for the lesion depths, the average lesion depth of negative control group was 172.91±21.97 μm, and of groups treated with 0.08 mg/mL, 0.12 mg/mL, 0.16 mg/mL rGO/Ag were 151.03±18.13 μm, 115.25±6.84 μm, 99.82±17.04 μm. The average lesion depths of groups treated with rGO/Ag were significantly smaller than the negative control group (p<0.05). The lesion depth of the 0.16 mg/mL AgNPs group (140.41±16.45 μm) also had significantly reduced depth (p<0.05) and no obvious lesion formation was observed in the 10 ppm NaF group.

### Degree of demineralized enamel layer

**Figure 5:**
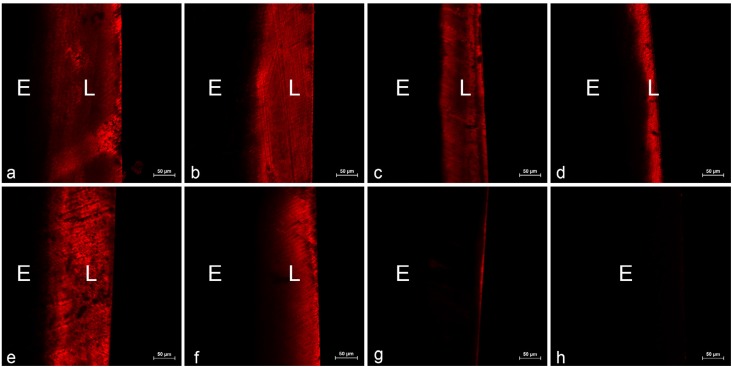
Cross-sectional confocal laser scanning microscopy (CLSM) images of the specimens after each treatment (magnified at 200x; L: lesion; E: intact enamel). (a) negative control; (b) 0.08 mg/mL rGO/Ag; (c) 0.12 mg/mL rGO/Ag; (d) 0.16 mg/mL rGO/Ag; (e) 0.16 mg/mL rGO; (f) 0.16 mg/mL AgNPs; (g) 10 ppm NaF; (h) native enamel

**Figure 6:**
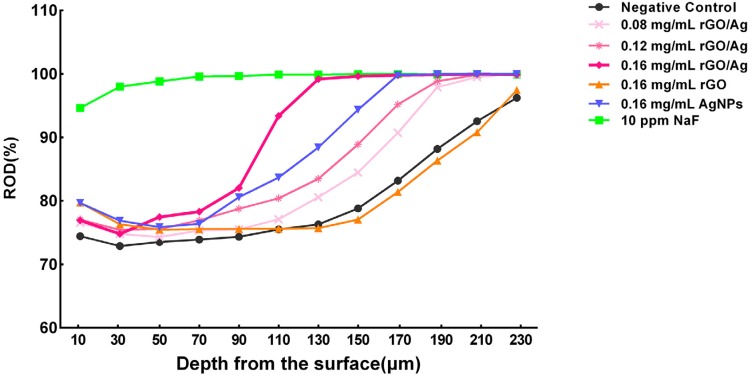
Profiles of each group and relative optical density (ROD) vs. depth (mm) for all the corresponding treatment groups

Representative CLSM images of the cross section of specimens from each group are shown in Figure 5 . After formation of enamel carious lesion, the samples were stained with rhodamine B, and a red fluorescent band, indicating the demineralized enamel layer, was observed on the superficial layers of the specimens in the treated groups. It was apparent that the demineralized layer depth differed among the groups treated with rGO/Ag or AgNPs, and negative control groups. The narrowest fluorescent band was observed in the group treated with 0.16 mg/mL rGO/Ag. No obvious artificial enamel carious lesions were observed in the 10 ppm NaF group. Table 1 shows the relative optical density (ROD) values of each treatment group at different sites of the lesion. Groups treated with rGO/Ag were able to induce a significant increase in the relative optical density (ROD) compared with the negative control group, indicating less tissue porosity. Especially in the 0.16 mg/mL rGO/Ag group, the ROD value was significantly higher at the lesion (depth=50 μm) when compared with the negative control group (p<0.05). Moreover, no significant differences in the ROD values were detected between the 0.16 mg/mL rGO/Ag and the 10 ppm NaF groups, at the body of the lesion (depth=130 μm; p=0.656). Representative profiles of the ROD values across the enamel lesions from the surface to deep enamel are shown in Figure 6 .

**Table 1:** Relative optical density (ROD) of artificial enamel lesions treated with rGO/AG, GO and AgNPs at different depths of tooth specimens (n=10)

Caries depth	Negative Control	0.08 mg/mL rGO/Ag	0.12 mg/mL rGO/Ag	0.16 mg/mL rGO/Ag	0.16 mg/mL rGO	0.16 mg/mL AgNPs	10 ppm NaF
10 μm	74.44±3.35^c^	76.58±3.30^bc^	77.06±1.66^bc^	76.93±3.47bc	79.73±6.16^b^	79.69±7.54^b^	94.64±3.87^a^
30 μm	72.89±2.73^c^	74.80±3.49^bc^	75.43±2.90^bc^	74.81±3.85bc	76.33±3.41^b^	76.87±3.98^b^	97.99±2.34^a^
50 μm	73.53±2.28^c^	74.32±2.81^c^	75.59±3.18^bc^	77.47±5.08^b^	75.46±3.54^bc^	75.86±3.68^bc^	98.84±2.26^a^
70 μm	73.92±2.04^c^	75.33±3.29^bc^	76.94±4.74^bc^	78.30±5.17^b^	75.71±3.24^bc^	76.40±4.51^bc^	99.60±0.79^a^
90 μm	75.35±3.49^c^	75.51±3.65^c^	78.76±6.18^bc^	82.04±6.19^b^	75.61±3.62^c^	80.59±5.65^b^	99.50±0.85^a^
110 μm	75.51±3.71^d^	77.11±3.85^d^	82.41±3.85^c^	93.40±3.86^b^	75.57±3.78^d^	83.69±3.63^c^	99.89±0.55^a^
130 μm	76.31±4.45^c^	75.59±3.84^d^	83.50±3.47^b^	99.20±1.20^a^	75.79±3.84^c^	86.42±5.01^b^	99.91±0.37^a^
150 μm	78.82±4.07^b^	84.48±3.61^bc^	85.90±3.26^c^	99.64±0.64^a^	77.07±3.58^b^	94.37±5.08^ac^	99.98±0.21^a^
170 μm	86.18±9.82^b^	90.73±7.46^bc^	95.20±5.97^cd^	99.81±0.39ac	81.40±10.78^b^	99.82±0.33^ac^	100.00±0.20^ad^
190 μm	90.20±8.55^b^	97.91±2.38^bc^	98.85±1.35^c^	99.87±0.28ac	86.37±11.08^b^	100.00±0.06^ac^	99.95±0.20^ac^
210 μm	92.55±8.94^b^	99.51±0.66^bcd^	99.87±0.31^ac^	99.98±0.12ac	90.81±9.64^bc^	100.00±0.09^ad^	99.98±0.14^ac^
230 μm	96.25±5.08^a^	100.00±0.06^bc^	100.00±0.07^bc^	99.89±0.25ac	97.47±2.30^ac^	100.00±0.07^bc^	99.90±0.25^ac^

ROD was calculated as ODR=ODl/ODs × 100%; ODl represents the OD of the enamel demineralized layer, ODs represents the OD of the sound enamel tissue (blank control) measured at the corresponding tooth specimen level. Values annotated with different superscripts (a–d) within the same row indicate statistically significant differences (p<0.05) between different groups

## Discussion

According to research on caries, the control of dental plaque formation is an important prevention strategy. One of the most effective methods for controlling dental plaque is the application of antimicrobial agents. Novel nanomaterials with a wide range and steady antibacterial properties can be used as promising cariostatic agents. ^10^ ^,^ ^18^ Graphene oxide and silver nanoparticles composites with high and long-term antibacterial effects are the most popular nanomaterials used in recent years. It displayed good cytocompatibility and its cytotoxicity was lower than that of AgNPs. ^11^ Researchers have reported that graphene oxide and silver nanoparticles composites show excellent antibacterial effects against Grampositive *S. aureus* and Gram-negative *Escherichia coli.* ^12^ Reduced graphene-silver nanoparticles (R-GNs/Ag) composite exhibited higher antibacterial activity against oral pathogens when compared with AgNPs, the stronger antibacterial properties of graphene oxide and silver nanoparticles composite are attributed to the efficient dispersion of AgNPs on graphene oxide sheets. ^13^ Meanwhile, graphene oxide can trap bacteria on its matrix and provide sites with high Ag^+^ concentration. ^19^ However, there was no available experimental data on the anticariogenic potential of the rGO/Ag composite. The aim of this study was to evaluate the inhibitory effect of rGO/Ag composite on the progression of artificial enamel caries in a *S. mutans* biofilm model.

*S. mutans* , which is considered as the main cariogenic bacteria, plays an important role in the initiation and progression of dental caries. ^20^ *S. mutans* microbial biofilm-induced caries model is an effective approach to investigate the protective property of antibacterial agents in enamel demineralization, and can better simulate the oral environment when compared with *in vitro* pH-cycling models. ^16^ To investigate the inhibitory effect of rGO/Ag composite on dental caries, this study produced artificial enamel carious lesions by allowing the formation of a *S. mutans* biofilm on bovine teeth specimens for 7 days. Following, the microstructure of the enamel surface and enamel lesion was observed, and the surface roughness of the caries enamel, the lesion depth of enamel lesion, the relative optical density (ROD) of the demineralized layer were measured to evaluate the severity of enamel caries.

To fully characterize the artificial enamel caries surface, multi-technical approaches, including 3D Non-Contact Optical Profile and AFM imaging, were employed in this study. 3D Non-Contact Optical Profile technique is performed to measure the surface roughness and it provides a more precise method to quantitatively characterize the surface topography nondestructively. ^21^ Surface roughness plays an important role in plaque accumulation, which reflects the bacterial adhesion potential and the dental carious lesion severity. ^22^ According to the measurements of enamel caries surface roughness, the Ra and Rq of specimens in the groups treated with the rGO/Ag composite was significantly decreased compared with the negative control group (p<0.01), indicating reduction of acid production and artificial enamel lesions. The AFM technique has a high-resolution capacity and is used to accurately assess the surface morphology by providing more intuitive three-dimensional micrographs at the nanometer scale. ^23^ In this study, the enamel specimens were preserved in sterile artificial saliva at 37°C, which simulates the normal oral conditions. The enamel surface of specimens in the negative control group displayed rough areas, visible voids, and cracks. These observations conformed to the principal histological features of naturally-occurring caries and indicated that the formation of enamel caries was successfully simulated. Notably, the enamel surface of specimens in the 0.16 mg/mL rGO/Ag group was less eroded and much smoother than in the control and the other treatment groups. This result suggested that the carious lesions were still in the primary stage. Based on the observations of the morphology of enamel caries surface and the results of surface roughness, the samples in the rGO/Ag groups exhibited lower enamel surface, surface roughness, and less eroded surfaces, relative to the specimens in the control and the other composite treatment groups.

As a standardized method for research on teeth demineralization, polarized light microscopy is widely used in the histopathological study of dental caries. ^24^ According to the morphology of the enamel lesions of samples from the negative control group, the *S. mutans* biofilm model showed the intact mineralization surface and body of the lesion, corroborating the principal histological features of natural caries. The enamel lesion depth of the negative control group was 172.91±21.97 μm, which was consistent with the research of lesion depth in 7-day *S. mutans* biofilm enamel caries model studies. ^16^ They indicated that the *S. mutans* biofilm enamel caries model was successfully constructed. Transversal microradiography (TMR) is the gold standard to characterize the demineralization, ^25^ but the mineral will reduce during sample preparation and the dental tissue will change in the measurement. ^24^ CLSM, which can quantify the fluorescence emitted by the rhodamine B dye, is generally considered as the most sensitive method to measure the extent of enamel demineralized layer. ^26^ Enamel carious lesion is caused by mineral loss, which allows the fluorescent dye to penetrate into the pores of the demineralized enamel. Therefore, the intensity and width of the fluorescent layer represents the depth of the demineralized layer. ^27^ In the groups treated with the rGO/Ag composite, the red fluorescent band was narrower compared with the negative control group, and the reduction of the band width was dose-dependent. The results of the demineralized layer depth were in agreement with the observations on enamel caries surface and carious lesions. In the group treated with 0.16 mg/mL rGO/Ag, the depth of enamel carious lesions was about 99.82±17.04 μm and the ROD values at different depths of the specimens were significantly higher than those in the negative control group. In contrast, the carious lesion depth and the ROD values of the 0.16 mg/mL rGO group had no significant differences with those of the negative control group. According to the published literature, the antibacterial property of graphene materials is still controversial. Gurunathan, et al. ^28^ (2012) reported that rGO showed time- and dose-dependent antibacterial activity. On the contrary, it was also reported that graphene oxide lacked bacteriostatic activity and enhanced bacterial growth by increasing their attachment surface and inducing bacterial proliferation. ^29^ Therefore, rGO may act as a general enhancer of caries progression by promoting *S. mutans* growth, which could explain its insufficiency to prevent formation of enamel caries in our study. Moreover, in the group treated with 0.16 mg/mL AgNPs, the tooth specimens displayed deeper carious lesion and lower ROD values compared with those in the group treated with 0.16 mg/mL rGO/Ag. In conclusion, the samples in groups treated with the rGO/Ag composite exhibited shallower lesion depths and higher ROD values, which indicated less mineral loss, compared with the rGO and the AgNPs treated groups.

Based on the results of this study, the rGO/Ag composite has a promising anticariogenic activity, and it can potentially be incorporated into the existing dental materials, as glass ionomer cements, dental resin composites, adhesive and denture resin bases, aiming at preventing secondary caries. Meanwhile, rGO/Ag composite with excellent antibacterial activity may also be used in root canal irrigation and disinfection, as well as surface modification of dental implant to reduce the chance of peri-implantitis. However, most of these promising results were achieved under mono-species biofilm model. To apply this novel composite material for caries prevention, the preventive effect of rGO/Ag composite on progression of caries in multi-species biofilm model needs to be confirmed. The long-term antibacterial effect of the use of rGO/Ag as additive to modify dental materials remains to be investigated.

## Conclusions

Collectively, the results of this study demonstrate that rGO/Ag has a protective role on enamel caries progression. This suggests the potential applications of rGO/Ag as a novel composite material for caries prevention.
